# Bibliographical

**Published:** 1883-06

**Authors:** 


### Bibliographical.

---

OUR BOOK TABLE.

There are certain journals which no professional man can afford to do without. *The Century Magazine* is one of these. Established by the late Charles Scribner, and edited for years by the lamented Dr. Holland, it rapidly won its way to popular favor and became pre-eminent in the world of letters, as in that of art. In the latter department, in fact, it may be said to have wrought a revolution by its wondrous specimens of wood engraving. Under the conduct of its present managers it has taken higher ground than ever before, and may now justly be rated as first in its class. Every complete office has *The Century* upon its center table.

The physician who desires a complete epitome of whatever is new in medicine, the freshest thought in all its departments, needs *The Medical Record*, a weekly, published by the house of Wm. Wood & Co. It is emphatically a *Record* of all that is taught and achieved in medicine. It is important alike to the dentist and the regular practitioner, if either would keep abreast the advance of thought in all departments of medicine.

*Popular Science Monthly*, published by D. Appleton & Co., is another of the journals indispensable to every man who desires to know what is going on in the world of thought. It has contained some of the most valuable original contributions which have been made to modern science. A glance at the table of contents of the June number makes one wonder how he has ever existed without it. Every medical man will certainly wish to read Dr. Wilders' "Vivisection in the State of New York," as well as Dr. Shepherd's "Medical Quacks and Quackeries."

The *New York Sanitarian* is especially devoted to the preservation of health, and to mental and physical culture, and is always full of suggestive articles that ought to be read by everybody. It is not only of special import to the professional man, but it is entertaining to the general reader, while to every one who has a home and family it should be indispensable.

*Valedictory address delivered at the First Commencement Exercises of the Dental Department of the University of California*, by S. W. DENNIS, M. D., D. D. S., Dean of the Faculty and Professor of the Principles and Practice of Operative Dentistry, and Dental Histology—and by H. J. PLOMTEAUX, D. D. S.

We congratulate the dental profession of the Pacific slope, that at last they have an educational institution of their own. The addresses under consideration are mainly devoted to an exposition of the course of study and the advantages and prospects of that school. We hope it may be as successful as its best friends can desire.

*Notes on Operative Dentistry*; by MARSHALL H. WEBB, D. D. S. Philadelphia; The S. S. White Dental Manufacturing Co., 1883.

The profession has not yet ceased to mourn for the loss of Dr. Webb, nor will it do so for some time, we hope. So genial, so kindly in disposition, so eminent as an operator, it was a severe blow to professional progress when he was removed. It is sad to reflect that all there is left of him to-day is the kindly personal recollections of his presence, the remembrance of his faithful teachings, and this book of one hundred and seventy-five pages, containing a record of the life-work of its author.

We cannot give our readers a better idea of its scope than by publishing its table of contents, merely saying that every chapter is an epitome of the ideas upon that subject so earnestly urged by Dr. Webb during his life.

Chap. 1. Histology.
" 2. The Deciduous Teeth—Prevention of Irregularity and Decay.
" 3. Application of the Rubber Dam.
" 4. Preparation of Filling materials.
" 5. The Mallet.
" 6. Filling cavities in masticating surfaces.
" 7. Filling cavities within Labial and Buccal Walls.
" 8. Pieces of Porcelain for Filling Cavities of Decay.
" 9. Filling cavities within Approximal Walls.

" 10. Preparation of Cavities for Restoration of Contour.
" 11. Restoration of Contour, and prevention of extension of Decay.
" 12. Summary of principles relating to Filling Teeth.
" 13. Covering and Protecting frail walls of Enamel with gold.
" 14. Placing Crowns on roots of Teeth.
" 15. Attaching Crowns to Teeth when Roots are Missing.
" 16. Irritation and death of the Pulp.
" 17. Filling Pulp-Chambers.
" 18. Treatment of Abscess.
" 19. Pericementitis.
" 20. Necrosis.

Every line written by Dr. Webb is worthy the most careful study, and to every portion of the book for which he is responsible we can give unhesitating commendation.

But there is a feature of which we speak with great reluctance, and only at the promptings of what we consider a duty to the profession. Of late, in both medicine and dentistry, the publishers of books have resorted to unjustifiable means to pad out the work, and at the same time to place their wares before the profession. Advertising cuts of goods and implements quite unnecessary to an understanding of the text are inserted, and thus an important subject is made puerile, while the whole work is tainted with a suspicion of the shop. Was it necessary, we would ask, to give an illustration of a common pair of forceps, of ordinary foil shears and plyers, of files and scrapers and excavators, that this book might be adapted to the comprehension of its readers? Every dental student is familiar with them, and their reappearance here, extracted from the catalogue of a dental manufacturing company, is scarce complimentary to the intelligence of the reader. They mar the book, and we are sincerely sorry that they were admitted. This is not the first dental book that has so offended good taste. Other publishers have done the same. The edition of Coleman, issued last year, looks like a dental catalogue, and other works have been open to the same criticism. We sincerely hope that the next book issued will not be defaced with these cheap cuts.

Webb's Operative Dentistry is handsomely printed and bound, and it contains an excellent portrait of its author, which of itself is almost worth the price of the book.

